# A very rare case of a small bowel leiomyosarcoma leading to ileocaecal intussusception treated with a laparoscopic resection: a case report and a literature review

**DOI:** 10.1186/s12957-016-0798-4

**Published:** 2016-02-24

**Authors:** Tomasz Guzel, Katarzyna Mech, Michał Mazurkiewicz, Bohdan Dąbrowski, Gustaw Lech, Andrzej Chaber, Maciej Słodkowski

**Affiliations:** Department of General, Gastroenterological and Oncologic Surgery, Medical University of Warsaw, ul. Banacha 1a, 02-097 Warsaw, Poland; Chair and Department of Pathomorphology, Medical University of Warsaw, ul.Pawińskiego 7, 02-097 Warsaw, Poland

**Keywords:** Small bowel tumour, Mesenchymal tumours, Leiomyosarcoma, Intussusception, Laparoscopy

## Abstract

**Background:**

Small bowel tumours are rare and comprise less than 2 % of all primary gastrointestinal neoplasms. Among these tumours, a leiomyosarcoma belonging to soft tissue sarcomas is extremely rare and accounts for about 1 % of malignant mesenchymal lesions in the gastrointestinal tract. Due to its aggressive nature and slow growth, it is often diagnosed at the late stage when curative treatment is impossible. Authors report a first case of leiomyosarcoma with chronic recurrent ileocaecal intussusception and literature review to analyse diagnosis and treatment features of the ileum mesenchymal tumours.

**Case presentation:**

We present a case of an 87-year-old Caucasian man suffering from cramp-like abdominal pain for months. Due to lack of clinical signs and unspecific complaints, a diagnosis was delayed. Despite a detailed in-hospital examination, a proper diagnosis was established as late as during an operation. The patient was treated by surgery with good results. An uncommon laparoscopic resection of the small bowel with a tumour was performed. A histopathological investigation confirmed a very rare mesenchymal lesion of the distal ileum. The patient is under control with no recurrence for 1 year of the follow-up period.

**Conclusions:**

Reported case indicates that a usually asymptomatic tumour can cause uncommon chronic recurrent ileus signs. CT and MRI scans are investigation of choice in such cases, but they are sometimes inconclusive. It might be worth highlighting the good results of laparoscopic leiomyosarcoma lesion resection with a very good outcome.

## Background

Abdominal pain is one of the most commonly reported ailments with no relation to the sex or age. It is often the first symptom of serious gastrointestinal tract diseases. Differentiation is difficult. Many of reported abdominal pain cases are caused by gastroenteritis or woman’s diseases. Sometimes, it can be caused by a very rare disease that shows unspecific signs and has to be well differentiated for curative treatment.

We present a very rare case of a patient with severe abdominal pain in the right lower quadrant caused by intussusception of the small bowel with a leiomyosarcoma in the caecum with ileocaecal subocclusion.

A leiomyosarcoma (LMS) is a rare smooth muscle malignant neoplasm which appears mostly in the retroperitoneal space, vascular wall (especially of the great veins) and soft tissues of the lower extremities. It is an exceptionally rare tumour of the gastrointestinal tract arising in such localisations like the oesophagus, stomach and small bowel. LMS belongs to a heterogeneous group of soft tissue neoplasms, and with its aggressive nature, it often presents local recurrence and visceral metastases. As in the presented case, clinical manifestation of this disease, as in other small bowel tumours, is unspecific. Authors report a very rare case of recurrent intussusceptions with tumour treated by laparoscopy with good results. To our best knowledge, there is only one published case of leiomyosarcoma treated by laparoscopic resection, but we have not found any papers concerning chronic recurrent intussusception caused by leiomyosarcoma.

## Case presentation

An 87-year-old Caucasian patient, suffering from arterial hypertension and hepatitis C, was admitted to the surgical department due to abdominal pain and weight loss of about 10 % during last 4 months.

For 6 months, the patient had been complaining about cramp-like abdominal pain in the right lower quadrant and suprapubic area, vomiting and bloody stools 3–4 times last week. He also noticed that eating, but not drinking, had exacerbated pain which usually stopped without using any drugs within 3–4 h. His past medical history revealed a stomach resection due to a peptic ulcer with relaparotomy due to bleeding immediately after an operation and cholecystectomy about 20 years ago.

**Fig. 1:**
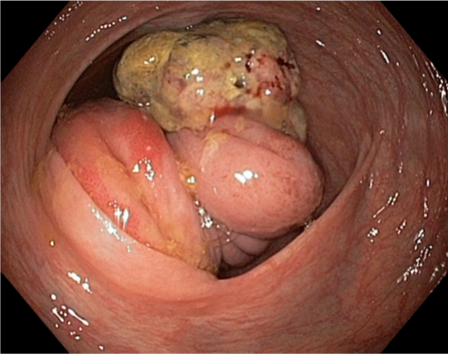
Jejunal tumour invaginated into the caecum—endoscopic view

On admission, the patient was in a good condition, with adequate logical responses and without complaints. No palpable mass in the abdomen was noted during an examination. Laboratory test revealed WBC 6250/uL, RBC 4,150,000/uL, PLT 263,000/uL, and low-grade anaemia Hb 11.8 g/dL. The cancer antigen levels were normal: carcinoembryonal antigen (CEA) 2.29 ng/mL (normal range 0.00–4.30 ng/mL) and CA 19-9 antigen 16.36 IU/mL (normal range 0.00–39.00 IU/mL). A computer tomography (CT) scan confirmed the presence of two suspected findings in the sixth and eighth liver segments, deep in the stroma. Results remained unclear, and magnetic resonance imaging (MRI) was performed with no conclusion. Neither CT scan nor MRI did not reveal any evidence of tumour and intussusception probably due to lack of occlusion signs at the moment. A colonoscopy performed revealed a caecal tumour, probably involving Bauhin’s valve (Fig. 1). A histopathological examination of samples collected confirmed the presence of unknown cancer cells.

**Fig. 2:**
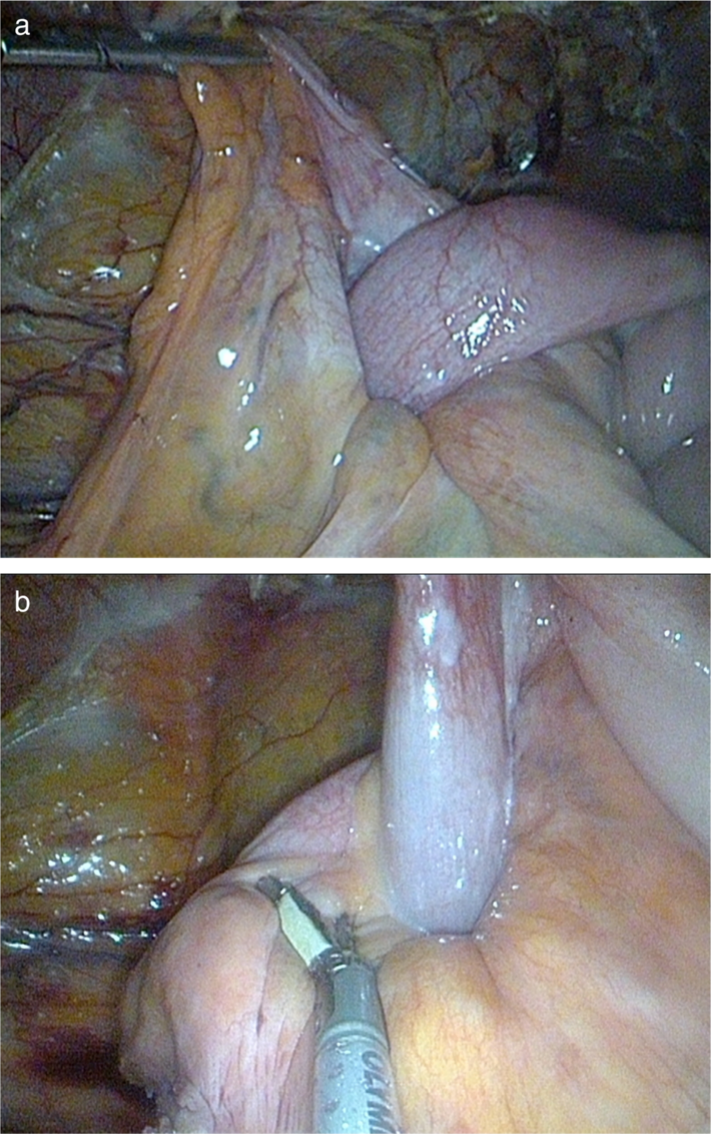
**a** Intussusception of the non-gangrenous small bowel into the caecum. **b** Intussusception of the non-gangrenous small bowel into the caecum

**Fig. 3:**
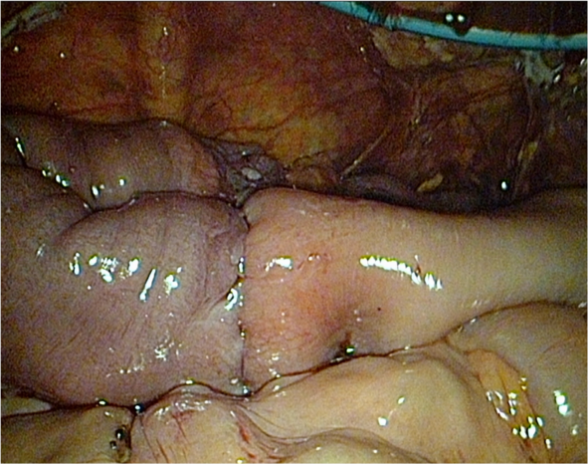
End-to-end anastomosis after resection—laparoscopic view

In accordance with a preoperative diagnosis (a caecal tumour), the patient was qualified for a laparoscopy in order to perform right hemicolectomy. During a surgery, intussusception of about 50 cm of the non-gangrenous small bowel into the caecum was found (Fig. 2a, b). Liver investigation did not reveal any signs of metastases as they were localised deep in stroma due to CT scan results. No other pathologies including enlarged lymph nodes and distant metastases were noticed. After bowel elongation, the segment with a tumour was resected and an extracorporal end-to-end anastomosis was performed (Fig. 3).

**Fig. 4:**
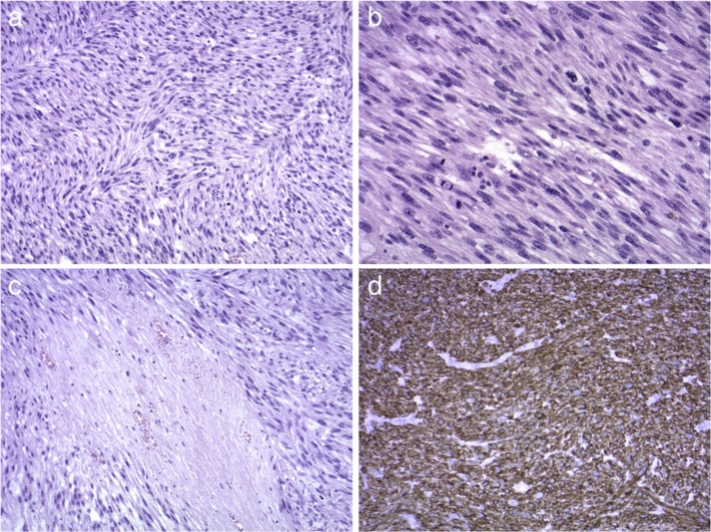
Histopathological results. **a** Spindle cell tumour with marked pleomorphism, HE stain, mag. ×100. **b** Brisk mitotic activity, HE stain, mag. ×200. **c** Focal necrosis, HE stain, mag. ×100. **d** Tumour shows positive immunoreactivity for smooth muscle actin

Postoperative histopathological results revealed a tumour of 5 × 4 × 3.7 cm, C-kit (−), CD34 (−), S100 (−), actin (+), high mitotic activity—28 figures per 10 HPFs, and high degree of necrosis (Fig. 4a–d).

The postoperative period was uneventful; no complications were noticed. The patient, after intensive postoperative rehabilitation, was discharged on day 7, in a good condition.

After a consultation with an oncologist, patient was disqualified from adjuvant therapy. Due to hepatitis C, advanced age and past surgical history, he was also not qualified for a liver metastases resection.

The 12-month follow-up period completed, and no recurrence signs were noticed. Liver metastases are stable in CT imaging tests.

Small bowel tumours (SBT) are rare and comprise less than 2 % of all primary gastrointestinal (GI) neoplasms [1, 2]. There are several conditions which seem to be associated with an increased risk for developing small bowel malignancies including inflammatory bowel disease—Crohn’s disease, polyposis syndromes, hereditary non-polyposis colorectal cancer (HNPCC) or caeliac disease. The highest morbidity for SBT is in the North America and Europe, related to men in majority. Twenty-five percent of all cases are malignant [2]. Both in the USA and Europe, the most common is carcinoid (37.4 %) and adenocarcinoma (36.9 %) that occurs two to three times more frequently than lymphangioma (17.3 %) and stromal tumours (8.4 %) [3].

LMS is a rare smooth muscle malignant neoplasm and belongs to soft tissue sarcomas (STS). In late 1990s, due to the introduction of new immunoassay tests, there were changes in the classification of GI mesenchymal tumours, and currently, most of them are classified as gastrointestinal stromal tumours (GIST). Primary leiomyosarcomas are diagnosed rarely. These are aggressive neoplasms often with severe cellular atypia and numerous mitoses. Definitive tools allowing for differentiation leiomyosarcoma from GIST are actin and desmin positive reactivity, CD117, CD34 and PDGFRA (platelet-derived growth factor receptor-alfa) negativity. According to 2013 Protocol for the Examination of Specimens from Patients With Gastrointestinal Stromal Tumor (GIST) that we based our diagnosis on, stainings listed above were done in the specimen obtained from our patient [4, 5] (http://www.cap.org/apps/docs/committees/cancer/cancer_protocols/2013/GIST_13protocol_3022.pdf). DOG1, chloride channel protein named “Discovered-On-GIST 1”, was published as a useful tool in GIST differentiation but was not listed in our local protocols in the time of diagnosis [6–9]. It was added to the list of immunohistochemical studies in recommendations published in 2014 by College of American Pathologists [9] (http://www.cap.org/ShowProperty?nodePath=/UCMCon/Contribution%20Folders/WebContent/pdf/cp-gist-14protocol.pdf).

The incidence of primary leiomyosarcoma in the gastrointestinal tract accounts for 0.1–3 % and that is fiftieth that of GIST [5, 10].

Many publications confirm rarity of the disease. In meta-analysis of the English literature in years 2002–2012 published by Aggarwal et al., authors noticed only 26 cases of small bowel leiomyosarcomas [4]. Also, after a retrospective analysis of 262 cases of GI mesenchymal lesions diagnosed in one centre in the period of 12 years, Agaimy et al. confirmed three cases of LMS which account for only 1.1 % [11].

Although most leiomyosarcomas are sporadic neoplasms, several aetiologic factors have been determined. LMSs more commonly occur in patients with a history of retinoblastoma, HIV infections, immunocompromised patients after transplantations and children with congenital immunodeficiency. Tumours have also been found to be associated with Epstein-Barr virus (EBV) as a nuclear antigen-2 protein and specific EBV receptor in smooth muscle cells are expressed in immunocompromised individuals [4].

Generally, STS prognosis is poor; the median overall survival is about 12 months [9]. A 5-year survival rate in patients with tumours over 5 cm in diameter and high mitotic activity is from 5 to 27 % [12, 13].

Several histologic grading systems have been validated in soft tissue sarcomas, including LMSs; but no system is currently accepted worldwide. According to the World Health Organisation (WHO), the grading of sarcomas should be performed using the US National Institute of Health (NIH) or French Federation Nationale des Centres de Lutte Contre le Cancer (FNCLCC) grading systems. The ‘French system’ evaluates three tumour parameters: a degree of tumour differentiation, number of mitotic figures per 10 HPFs and a degree of tumour necrosis. The American system uses a histological diagnosis coupled with a degree of cellularity, cellular polymorphism and mitotic activity. A degree of necrosis is also identified, and a result greater than 15 % diminishes the prognosis. It has been demonstrated that the FNCLCC results show a better correlation with overall and metastasis-free survival [4, 14].

Due to late clinical manifestation, tumours are diagnosed rather late when subileal or GI perforation has already developed. Permanent bleeding into the gastrointestinal tract has to be confirmed by a faecal occult blood test as there is usually no massive haemorrhage. Nausea, vomiting, flatulence and abdominal pain are often devaluated and delay a diagnosis [2].

In the presented case, the main complaint was recurrent abdominal pain diagnosed by a general practitioner (GP) as gastroenteritis and treated with a muscle relaxant and stomach protective drugs. There were no signs of a subileal or abdominal mass on palpation; therefore, a GP decided to send the patient for further tests when no recovery was observed. Intussusception was not suspected.

It is worth mentioning that intussusception was described for the first time in 1674, and successful operation in a child was performed in 1871 [15]. This disease is the most common in infants between the age of 5 and 10 months. It is the second leading cause of ‘acute abdomen’ in children following appendicitis. Morbidity in adults is estimated to be around two to three cases per 1,000,000 per year. Intussusception accounts for about 1 % of ileal and 0.08 % of all surgical interventions. Otherwise, 60 % of all adult patients suffering from the disease require emergency operation [16]. Intussusception is usually caused by neoplasms, adhesions or diverticula [16, 17]. A physical examination is usually difficult. Ultrasonography and computer tomography seem to be radiological tests of choice, with CT scan sensitivity of about 70–88 % and specificity of 100 % [16, 18]. Despite this, a proper preoperative diagnosis is made in only about 35–50 % of all cases with intussusception confirmed latter by a laparoscopy or laparotomy [19–21].

With regard to our imaging tests, both CT and MRI scans did not confirm any diagnosis, and the only findings included suspected metastases in the liver. It is worth highlighting that, due to clinical signs before admission, probably, intussusception and suboclusion occured and reduced spontaneously many times what caused investigation difficulties. A colonoscopy confirmed the presence of a tumour, but wrong localisation on Bauhin’s valve was described. Histopathological findings from colonoscopy samples were also not diagnostic with regard to a type of a neoplasm. The patient was qualified for a laparoscopy in order to perform right hemicolectomy as a caecal tumour was suspected in a preoperative examination. During a surgery, when intussusception of the ileum into the caecum was diagnosed and a small bowel tumour was confirmed, we had to change an operating procedure into an ileal resection.

Laparoscopy procedures in case of smooth muscle neoplasms are rare. As a result of searching the PubMed database, only one case of a segmental laparoscopic resection of the small bowel due to a leiomyosarcoma described by Hamm et al. in 2013 was found [22]. These neoplasms rarely give lymph nodes metastases, and advanced lymphadenectomy in these operations is usually not required. It is possible to perform minimally invasive treatment such as a laparoscopy instead of laparotomy [1, 13]. A laparoscopy in GI tumours used to be performed as a safe and acceptable method of colorectal cancer (CRC) treatment. Between 1997 and 2003, the COLOR study was conducted in seven EU countries (COLon cancer Laparoscopic or Open Resection) involving 29 hospitals. There were no differences in an open versus laparoscopic approach with regard to tumour staging, localisation, diameter, histological type, lymph nodes resected/metastasised and R1/R2 resection (microscopic/macroscopic non-curative resection) [23]. Another multicentre study (48 centres) involving over 870 patients suffering from CRC confirmed the same recurrence rate for both types of a surgery [24]. A retrospective analysis of over 430 patients operated at the Cleveland Clinic between 1996 and 2007 revealed the same rate of all resected lymph nodes and a higher rate of metastasised lymph nodes after a laparoscopic resection compared to an open access [25].

When compared to the results of other reports concerning colon cancer treatment, our report demonstrates that a laparoscopic resection of small bowel sarcomas may be a reasonable and safe alternative to a usually performed open resection.

As it was already mentioned with regard to current histopathological classification, smooth muscle neoplasms are very rare—gastrointestinal stromal tumours are predominantly diagnosed. Proper differentiation between these two types of STSs is very important to select appropriate adjuvant therapy. In some cases, leiomyosarcomas can be treated by conventional chemotherapy, whereas GIST therapy requires different drugs such as imatinib—a tyrosine kinase inhibitor [4, 26]. In LMS treatment, efficacy of adjuvant chemotherapy is limited. Tumours are also resistant to radiotherapy [1]. With regard to the most active drugs used in STS therapy, namely doxorubicin and ifosfamide, the response rates around 15–20 % can be achieved. New agents, such as trabectedin with gemcitabin or pazopanib, are unsatisfactory yet. Currently, there are no recommendations whether to use these agents in STS treatment [27].

## Conclusions

Chronic recurrent cramp-like abdominal pain can be caused by serious gastrointestinal disease and often requires endoscopic investigation, especially in elderly patients. Intussusception in these cases might not be permanent; it occurs and reduces spontaneously but recurs. This can cause diagnostic problems and delays final diagnosis. A surgical resection, also by laparoscopy, is the treatment of choice and allows a patient to benefit from adjuvant therapy.

### Consent

Written informed consent was obtained from the patient for publication of this case report and any accompanying images. A copy of written consent is available for review by the Editor of the journal.

### Confirmation

The authors confirm that the content of the manuscript has not been published, or submitted for publication elsewhere.

### Ethics

This case report is a retrospective study to report some important data concerning investigation and treatment of the patient. Informed consent was obtained. No ethics is required for study.

## Abbreviations

GI: gastrointestinal
LMS: leiomyosarcoma
SBT: small bowel tumours
STS: soft tissue sarcomas

## References

[1] Samaiya A, Deo SVS, Thulkar S, Hazarika S, Kumar S, Parida DK (2005). An unusual presentation of a malignant jejunal tumor and a different management strategy. World J Surg Oncol.

[2] Paski SC, Semrad C (2009). Small bowel tumours. Gastrointest Endoscopy Clin N Am.

[3] Bilimoria K, Bentrem D, Wayne JD, Ko CY, Bennett CL, Talamonti MS (2009). Small bowel cancer in the United States—changes in epidemiology, treatment and survival over the last 20 years. Ann Surg.

[4] Aggarwal G, Sharma S, Zheng M, Reid MD, Crosby JH, Chamberlain SM (2012). Primary leiomyosarcomas of the gastrointestinal tract in the post-gastrointestinal stromal tumor era. Ann Diagn Pathol.

[5] Yamamoto H, Handa M, Tobo T, Setsu N, Fujita K, Oshiro Y (2013). Clinicopathological features of primary leiomyosarcoma of the gastrointestinal tract following recognition of gastrointestinal stromal tumours. Histopathology.

[6] Lee CH, Liang CW, Espinosa I (2010). The utility of discovered on gastrointestinal stromal tumor 1 (DOG1) antibody in surgical pathology—the GIST of it. Adv Anat Pathol.

[7] Swalchick W, Shamekh R, Bui MM (2015). Is DOG1 immunoreactivity specific to gastrointestinal stroma tumor?. Cancer Control.

[8] Seo HS, Hyeon JY, Shin OR (2015). C-kit-negative gastrointestinal stromal tumor in the stomach. J Gastric Cancer.

[9] Chunwei X, Hongyan H, Jingjing W (2015). Diagnosis value of CD117 and PDGFRA, alone or in combination DOG1, as biomarkers for gastrointestinal stromal tumors. Ann Transl Med.

[10] Weledji EP, Enoworock G, Ngowe MN (2014). Gastric leiomyosarcoma as a rare cause of gastric outlet obstruction and perforation: a case report. BMC Res Notes.

[11] Agaimy A, Wünsch PH (2007). True smooth muscle neoplasms of the gastrointestinal tract: morphological spectrum and classification in a series of 85 cases from a single institute. Langenbecks Arch Surg.

[12] Licht JD, Weissmann LB, Antman K (1988). Gastrointestinal sarcomas. Semin Oncol.

[13] O’Riordan BG, Vilor M, Herrera L (1996). Small bowel tumors: an overview. Dig Dis.

[14] Jo VY, Fletcher CD (2014). WHO classification of soft tissue tumours: an update based on the 2013 (4th) edition. Pathology.

[15] Sarma D, Praghu R, Rodrigues G (2012). Adult intussusception: a six-year experience at a single center. Ann Gastroenterol.

[16] Potts J, Samaraee A, El-Hakeem A (2014). Small bowel intussusception in adults. Ann R Coll Surg Engl.

[17] Lianos G, Xeropotamos N, Bali C, Baltoggiannis G, Ignatiadou E (2013). Adult bowel intussusception: presentation, location, etiology, diagnosis and treatment. G Chir.

[18] Manouras A, Laqoudianakis E, Dardamanis D, Tsekouras D, Markoqiannakis H, Genetzakis M (2007). Lipoma induced jejunojejunal intussusception. Word J Gastroenterol.

[19] Barussaud M, Regenet N, Briennon X, de Kerviler B, Pessaux P (2006). Clinical spectrum and surgical approach of adult intussusceptions: a multicentric study. Int J Colorectal Dis.

[20] Azar T, Berger DL (1997). Adult intussusception. Ann Surg.

[21] Nagorney D, Sarr M, Mcllrath D (1981). Surgical management of intussusception in the adult. Ann Surg.

[22] Hamm JK, Chaudhery SI, Kim RH (2013). Laparoscopic resection of small bowel sarcoma. Surg Laparosc Endosc Percutan Tech.

[23] Veldkamp R, Kuhry E, Hop WC, Jeekel J, Kazemier G, Bonjer HJ (2005). Laparoscopic surgery versus open surgery for colon cancer: short-term outcomes of a randomized trial. Lancet Oncol.

[24] Clinical Outcomes of Surgical Therapy Study Group (2004). A comparison of laparoscopically assisted and open colectomy for colon cancer. N Engl J Med.

[25] El-Gazzaz G, Hull T, Hammel J, Geisler D (2010). Does a laparoscopic approach affect the number of lymph nodes harvested during curative surgery for colorectal cancer?. Surg Endosc.

[26] Lech G, Korcz W, Kowalczyk E, Guzel T, Radoch M, Krasnodębski IW (2015). Giant gastrointestinal stromal tumour of rare sarcomatoid epithelioid subtype: case study and literature review. World J Gastroenterol.

[27] Kasper B, Reichardt P, Pink D, Sommer M, Mathew M, Rauch G (2015). Combination of trabectedin and gemcitabine for advanced soft tissue sarcomas: results of a phase I dose escalating trial of the German Interdisciplinary Sarcoma Group (GISG). Mar Drugs.

